# Breast Meat Fatty Acid Profiling and Proteomic Analysis of Beijing-You Chicken During the Laying Period

**DOI:** 10.3389/fvets.2022.908862

**Published:** 2022-06-15

**Authors:** Jian Zhang, Hong Zhuang, Jing Cao, Ailian Geng, Haihong Wang, Qin Chu, Zhixun Yan, Xiaoyue Zhang, Yao Zhang, Huagui Liu

**Affiliations:** ^1^Institute of Animal Husbandry and Veterinary Medicine, Beijing Academy of Agriculture and Forestry Sciences, Beijing, China; ^2^United States Department of Agriculture, Agricultural Research Service, U.S. National Poultry Research Center, Athens, GA, United States

**Keywords:** fatty acid, Beijing-You chicken, pectoralis major, laying period, TMT-based quantitative proteomic analysis, parallel reaction monitoring

## Abstract

The disparity in fatty acids (FA) composition exhibits a significant impact on meat quality, however, the molecular regulatory mechanisms underlying this trait in chicken are far from clear. In this study, a total of 45 female Beijing-You chicken (BYC) hens, fed on the same diet, were collected at the slaughter age of 150, 300, or 450 days (D150, D300, and D450) from sexual maturation stage to culling stage (15 birds per age). Gas chromatography-mass spectrometry (GC-MS) and tandem mass tag labeling technology based on liquid chromatography mass spectrometry (TMT-LC-MS/MS) analysis strategies were applied to profile FA compositions and to compare differential expressed proteins (DEPs) between these different slaughter ages, respectively. The FA profiling showed that increasing hen ages resulted in increased contents of both saturated and unsaturated fatty acids. Proteomic analyses showed a total of 4,935 proteins in chicken breast muscle with the false discovery rate (FDR) < 1% and 664 of them were differentially expressed (fold change > 1.50 or < 0.67 and *P* < 0.01). There were 410 up- and 116 down-regulated proteins in D150 vs. D300 group, 32 up- and 20 down-regulated in D150 vs. D450 group, and 72 up- and 241 down-regulated in D300 vs. D450 group. A total of 57 DEPs related to FA/lipid-related metabolisms were obtained according to the enrichment analysis of Gene Ontology (GO) and Kyoto Encyclopedia of Genes and Genomes (KEGG). These DEPs were involved in 21 significantly enriched (*P* < 0.05) pathways, including well-known pathways for FA synthesis (metabolism, desaturation, and elongation) and the signaling pathways for lipid metabolism (PPAR, adipocytokine, calcium, VEGF, MAPK, and Wnt). In addition, there existed several representative DEPs (FABP, FABP3, apoA-I, apoA-IV, apoC-III, apoB, VTG1, and VTG2) involved in the regulation of FA/lipid transportation. The construction of the interaction networks indicated that HADH, ACAA2, HADHA, ACSL1, CD36, CPT1A, PPP3R1, and SPHK1 were the key core nodes. Finally, eight DEPs were quantified using parallel reaction monitoring (PRM) to validate the results from TMT analysis. These results expanded our understanding of how the laying age affects the FA compositions and metabolism in hen breast meat.

## Introduction

Fatty acid (FA) composition has been associated with not only meat quality, but also human health (1–4). It has been well known that monounsaturated fatty acid (MUFA) and polyunsaturated fatty acid (PUFA), especially n-3 FAs, are generally considered to have beneficial health effects (5). In contrast, the higher intake of saturated fatty acid (SFA) in the human diet increases the risk of the development of several chronic diseases, such as coronary heart disease (6), atherosclerosis (7), and cancer (8). In meat, the FA composition plays a key role in the texture, color, flavor, and oxidative stability (9). Moreover, the FA composition affects flavor development during cooking through the production of volatiles due to the FA oxidation (10, 11). Poultry is the second most-consumed meat worldwide and relatively affordable in most countries. Poultry meat provides the human with the essential PUFA, especially the n-3 FAs (12, 13). Hence, there has been a considerable interest in recent years in identifying the FA profiles and exploring the mechanism of the FA modulating in poultry meat as an alternative source of n-3 FA in the replacement of sea food, which is the primary source of PUFA in the human consumption.

Studies have suggested that the FA composition in poultry meat could be comprehensively modified through manipulating the feed diets, rearing system, genotype, and age (14–23). Among them, age is one of the most important factors. Dal Bosco et al. (17) demonstrated that older birds had higher long-chain PUFA as well as total PUFA levels. Popova et al. (20) showed the n-6/n-3 ratio decreased substantially in the older chickens. These results imply, to some extent, that aged chicken is tended to be more nutritional as well as flavorful, which, in fact, has made indigenous, slower-growing, and aged chickens very popular in some regions in the world (24, 25).

Recently, several studies have been carried out to elucidate intramuscular fat (IMF) deposition and meat quality traits in chickens through proteomics-based methods at some critical stages of development (26–31). However, these studies have mainly focused on IMF (26, 27) and abdominal fat (31, 32). Most of them were limited on embryonic muscle (28, 29) or during the bird body development stage (30, 31). To date, there was no report on the dynamic changes in FA composition and related proteins in chicken during the laying period, especially from the sexual maturation stage to the culling stage.

Beijing-You chicken (BYC), a famous Chinese indigenous dual-purpose breed, is characterized by excellent meat quality and slow-growing rate. In the present study, BYC was used as a model system to elucidate the variation of the FA compositons and the comparison of the differential expressed proteins (DEPs) due to the difference of the laying period. Gas chromatography-mass spectrometry (GC-MS) analysis strategies and a TMT-LC-MS/MS proteomics were applied to profile FAs compositions and determine the differentially expressed proteins (DEPs), respectively, in breast meat at 150, 300, and 450 d ages of laying, representing the early laying chronological age at sexual maturation (D150), the medium laying chronological age (D300) and late laying chronological age at culling stage (D450). All hen birds used in the study were fed under the same conditions to eliminate feeding interference. In addition, some candidate proteins were validated by the targeted parallel reaction monitoring (PRM) method. The results based on analyses of the DEPs would enhance our understanding of molecular mechanisms of FA metabolism during the chicken laying period, and pave the way for modulating FA in slow-growing poultry meat through the genetic method.

## Materials and Methods

### Ethical Approval

All of the animal experiments were conducted following the guidelines for experimental animals established by the Ministry of Science and Technology (Beijing, China). Animal experiments were reviewed and approved by the Science Research Department (in charge of animal welfare issues) of the Institute of Animal Husbandry and Veterinary Medicine, Beijing Academy of Agriculture and Forestry Sciences (Beijing, China), and the approval number was BAAFS-IAHVM20190115.

### Animals and Tissue Sampling

A total of 90 1-day-old female BYC birds, having the same genetic backgrounds, were obtained from the Institute of Animal Husbandry and Veterinary Medicine, Beijing Academy of Agriculture and Forestry Sciences. The hens were randomly divided into three groups and each group comprised 30 hens. Birds were raised in an environmentally controlled room with three floor pens under the recommended environmental and nutritional conditions for BYC. Diets were made from the same material sources according to the recommendations. Briefly, a three-phase feeding system (0–49, 49–120, and 120–450 d) was adapted in this study. For each phase, crude protein of diet was 19.00, 15.07, and 15.51%, respectively, and the metabolizable energy was 11.91, 11.20, and 11.08 MJ/kg, respectively. Birds were given *ad libitum* access to a standard diet and water throughout the whole rearing period. At each ages of 150, 300, and 450 d, a total of 15 birds were randomly selected, weighed, electrically stunned and killed by exsanguination, respectively. The average body weight of each laying age was 1,344 ± 63 g, 1,951 ± 266 g, and 2,228 ± 234 g, respectively. In addition, the average filet weight was 55.64 ± 7.68 g, 85.48 ± 10.93 g, and 113.20 ± 17.02 g, respectively. At each age, 10 meat samples (200 mg per piece) from the left filet (pectoralis major) of each bird were snap-frozen in liquid nitrogen, and stored at −80°C until the GC-MS and TMT-LC-MS/MS was carried out.

### Medium and Long Chain Fatty Acid Extraction

An accurate amount (200 mg) of chicken breast meat from each sample was weighed into 2 mL grinding tube with a small steel ball. One mL of chloroform: methanol (v/v = 1:1) mixture was added before the grinding tube was placed in a cryogenic grinding machine. The sample was ground at 50 Hz for 3 min, followed by ultrasonic for 15 min at a refrigerated temperature. The sample was let to stand at −20°C for 15 min, and then centrifuged at 13,000 × g and 4°C for 10 min. The supernatant was transferred into a 5.0-mL Eppendorf tube and dried with nitrogen. After that, 1.0 mL of methylation reagent (0.5 mol/L sodium hydroxide methanol solution) was added, followed by a 30 s vortex and incubation in a water bath at 60°C for 30 min. After cooling, 1.0 mL of n-hexane was added, followed by vortex for 30 s and centrifuge at 13,000 × g and 4°C for 10 min. The upper solution (n-hexane layer) was taken into a 1.5 mL centrifugation tube and dried with nitrogen. Finally, 200 μL n-hexane was added to the tube, followed by whirl ultrasound for 10 min and centrifugation (13,000 × g, 15 min, 4°C). The supernatant was carefully transferred to the sample vial for analysis.

### GC-MS Analysis

GC-MS analysis were conducted using an Agilent 8890B gas chromatography coupled to an Agilent 5977B mass selective detector with an inert electron impact ionization (EI) source. Ionization voltage was 70 eV (Agilent, USA). Analyte compounds were separated with an Agilent DB-FastFAME (20 m × 0.18 mm × 0.20 μm) capillary column (Agilent J&W Scientific, Folsom, CA, USA), using 99.999% helium as a carrier gas at a constant flow rate (1 mL/min). The GC column temperature was programmed to hold at 80°C for 30 s, and raise to 180°C at a rate of 70°C per min, then raise to 220°C at a rate of 4°C per min, finally hold at the temperature of 240°C for 2 min. The temperatures of the inlet and ion sources were 240 and 230°C, respectively. Temperature of the quadrupole was 150°C. The injection volume of the sample was 1 μL and introduced in splitting mode (50:1). Data acquisition was conducted on ion scan mode. Compounds were identified and quantified by the software of Masshunter (v10.0.707.0, Agilent, USA). The mass spectrum peak area of the analyte was used as the ordinate and the concentration of the analyte as the abscissa to draw a linear regression standard curve for calculation of FA contents in muscle.

### Indices of Fatty Acid Metabolism

Desaturase, elongase, and thioesterase activities of FAs in muscle tissue were estimated based on the ratio of product to the precursor of individual FA or the indices of FA metablism (16). The elongase index was calculated as the ratio of C18:0 to C16:0, and the thioesterase index was calculated as the ratio of C16:0 to C14:0 (33). The delta-9 desaturase index for the C16:1 and C18:1 was calculated according to the equation proposed by Haug et al. (34), which were calculated as the ratio of C16:1 to the C16:0 (SCD16) and the ratio of C18:1 to the C18:0 (SCD18), respectively. In addition, the index of delta-6 desaturase (D6D) was calculated as the ratio of C18:3n-6 to C18:2n-6, whereas the delta-5 desaturase (D5D) index was calculated as the ratio of C20:4n-4 and C20:3n-6 (34).

### Protein Extraction

An appropriate amount (100 mg) of chicken breast meat from each sample was frozen with liquid nitrogen and ground into a fine powder. Subsequently, the powder was suspended in 1.5 mL of protein lysis buffer (8M urea, 1% SDS), which contained appropriate protease inhibitor to inhibit protease activity. The mixture was incubated on ice for 30 min (during which the sample was vortexed 5–10 s every 5 min) and then sonicated for 2 min before centrifugation at 12,000 × g and 4°C for 20 min. The supernatant was collected, and the protein concentration was quantified using a BCA Protein Assay Kit (Thermo Scientific). Five extracts from each age stage were combined based on protein content (equal amounts) to have a total of 9 combined extracts (3 per age stage). The combined extracts were diluted to the same concentration with Tris-buffered saline (TBS) before protein digestion.

### Protein Digestion and TMT Labeling

A total of 100 μg protein from the combined extract was resuspended with triethylammonium bicarbonate buffer (TEAB) at the final concentration of 100 mM. Then the mixture was reduced with Tris (2-carboxyethyl) phosphine (TCEP) at the final concentration of 100 mM at 37°C for 60 min, followed by alkylation in iodoacetamide (IAM) at room temperature for 40 min (final concentration 40 mM) in the dark. Six-fold volumes of cold acetone were added to precipitate protein at −20°C for 4 h. The acetone mixture was centrifugated at 10,000 × g and 4°C for 20 min. The pellet was collected and resuspended with 100 μL of 100 mM TEAB. The protein suspension was digested with trypsin (the mass ratio of trypsin-to-protein at 1:50) overnight at 37°C. Trypsin-digested peptides were labeled with 10-plex TMT reagents according to the manufacturer's instructions (Thermo Fisher Scientific). Each sample was labeled with the corresponding TMT reagent. Briefly, after one unit of TMT reagent was thawed and reconstituted in 50 μL acetonitrile, the peptide mixture was incubated at room temperature for 2 h. Then hydroxylamine was added into the sample, and the mixture was incubated at room temperature for 15 min to quench the reaction. Finally, the multiplex labeled samples were pooled together in equal amounts before lyophilization.

### High pH RPLC Separation

The dried TMT-labeled peptides were resuspended with 5 mM ammonium hydroxide solution containing 2% acetonitrile. Subsequently, the samples were fractionated by Vanquish Flex binary UHPLC chromatography (Thermo, USA) with the ACQUITY UPLC BEH C18 Column (1.7 μm, 2.1 × 150 mm, Waters, USA) to increase proteomic depth. Briefly, peptides were first separated with a gradient of buffer B (5 mM ammonium hydroxide solution containing 80% acetonitrile, pH 10) at a flow rate of 200 μL/min over 48 min. The gradient was as follows: 0–1.9 min, buffer B maintained at 0%; 1.9–2 min, linear gradient from 0 to 5% buffer B; 2–17 min, buffer B maintained at 5%; 17–18 min, linear gradient from 5 to 10% buffer B; 18–35.5 min, linear gradient from 10 to 30% buffer B; 35.5–38 min, linear gradient from 30 to 36% buffer B; 38–39 min, linear gradient from 36 to 42% buffer B; 39–40 min, linear gradient from 42 to 100% buffer B; 40–44 min, buffer B maintained at 100%; 44–45 min, linear gradient from 100 to 0% buffer B; and 45–48 min, buffer B maintained at 0%. Eventually, 30 fractions were collected and then pooled into 15 fractions per sample.

### LC-MS/MS Analysis

The fractionated peptides were subjected to LC-MS/MS analysis and analyzed on a Q Exactive HF-X quadrupole orbitrap mass spectrometer coupled to an Easy nLC 1,200 (Thermo Fisher Scientific). Labeled peptides from each fraction were loaded onto a C18 reversed-phase column (75 μm × 25 cm, Thermo, USA), which were equilibrated and separated with buffer A (2% acetonitrile with 0.1% formic acid) and buffer B (80% acetonitrile and 0.1% formic acid) at a flow rate of 300 nL/min over 120 min. The peptides were eluted using the following gradient: 0–64 min, linear gradient from 5 to 23% buffer B; 64–80 min, linear gradient from 23 to 29% buffer B; 80–90 min, linear gradient from 29 to 38% buffer B; 90–92 min, linear gradient from 38 to 48% buffer B; 92–93 min, linear gradient from 48 to 100% buffer B; and 93–120 min, linear gradient from 100 to 0% buffer B. The Q Exactive HF-X instrument was operated in the data-dependent acquisition mode (DDA) to automatically switch between full scan MS and MS/MS acquisition. The survey of full scan MS spectra (350–1,500 m/z) was acquired in the orbitrap with 60,000 resolution at m/z 100. The automatic gain control (AGC) target value was 3.0 × 10^6^, and the maximum injection time was 20 ms. Then the top 20 most intense precursor ions were selected into collision cell for fragmentation by higher-energy collision dissociation (HCD). The MS/MS resolution was set at 45,000 at m/z 100. The AGC target at 2.0 × 10^5^ and the maximum injection time was 96 ms, and the dynamic exclusion was 30 s.

### Protein Identification

The resulting LC-MS/MS raw files were imported into ProteomeDiscoverer software (Thermo Scientific, version 2.4) for data interpretation and protein identification against the database UniProt-gallus gallus-34930-20200807.fasta (released in August 2020 and including 34,930 sequences). The search followed an enzymatic cleavage rule of trypsin/P and allowed two maximal missed cleavage sites and a mass tolerance of 20 ppm for fragment ions. The modification set was as follows: fixed modification: carbamidomethyl (C), TMT6plex (K), and TMT6plex (N-term); variable modification: oxidation (M) and acetyl (protein N-term). A minimum of 6 amino acids was required for peptides, and ≥ 1 unique peptide was required per protein. For peptide and protein identification, the false discovery rate (FDR) was set to 1%. The mass spectrometry proteomics data have been deposited to the ProteomeXchange Consortium *via* iProX partner repository (35) with the dataset identifier PXD031721.

### Data Statistics and Bioinformatics Analysis

The data were analyzed on the free online platform of Majorbio Cloud Platform (www.majorbio.com). The thresholds of fold change (FC) > 1.50 or < 0.67 and *P*-values <0.01 were set to identify DEPs. Annotation of all identified proteins was performed using GO (BLAST2GO, vision 2.5.0) and KEGG. GO and KEGG enrichment analyses were carried out with Fisher's exact test, and FDR correction for multiple testing was also performed. Enriched GO and KEGG pathways were nominally statistically significant at the *P* < 0.05 level. Protein-protein interaction (PPI) networks were also conducted using the STRING (version 11.0).

### Targeted Protein Quantification by LC-PRM/MS Analysis

To validate the TMT proteomics data, further liquid chromatography-parallel reaction monitoring MS (LC-PRM/MS) analysis was performed (30). PRM analysis was performed on a Q Exactive HF-X mass spectrometer (Thermo Fisher Scientific) and the optimized methods were generated experimentally using 1–3 unique peptides of high intensity and confidence for each target protein. The mass spectrometer was operated in positive ion mode with the following parameters: The full MS1 scan was acquired with a resolution of 60,000, with an AGC target value of 3.0 × 10^6^, and a maximum ion injection time of 20 ms. Full MS scans were followed by 39 PRM scans at a resolution of 15,000 with an AGC value of 5.0 × 10^5^ and a maximum injection time of 100 ms. The targeted peptides were fragmented at a normalized collision energy of 28 in a higher-energy collisional dissociation (HCD) cell. The raw data were analyzed using Skyline 4.1 (MacCoss Lab, University of Washington) to obtain the signal intensities of individual peptide sequences.

### Statistical Analysis

Data on FA composition and indices of FA metabolism were analyzed by the General Linear Model procedure of SAS (version 9.2, SAS Institute Inc., Cary, NC, USA). The three different chronological ages (150, 300, and 450 d) were analyzed as the main effect and the Tukey's method was used to identify significant differences between LSmeans (*P* < 0.05).

## Results

### Changes in FA Profiles of Breast Meat During Laying Period

The FA composition of the skinless breast meat of BYC was shown in Table 1. The SFA and MUFA at D450 (1,112.84 and 1,289 μg/g, respectively) were higher (*P* < 0.05) than those at D150 (619.79 and 647.74 μg/g, respectively) and D300 (690.37 and 709.38 μg/g, respectively), which exhibited no significant difference (*P* > 0.05). Moreover, the meat of D450 was characterized by a significantly higher (*P* < 0.01) concentration of total PUFA, both in the total content and in the different fractions (n-3 and n-6) when compared with D150 and D300. It was noticeable that the concentration of C20:5n3 (EPA) at D300 was significant lower (*P* < 0.01) than that at either D150 or D450, which did not differ from each other (*P* > 0.05); however no differences were detected in C22:6n3 (DHA), C20:3n6, or C20:4n6 (ARA) during the laying period (*P* > 0.05).

**Table 1:** Influence of laying age on the fatty acid profiles (μg/g of DM) of the skinless breast meat (mean ± SD, *n* = 10)^1^.

**Item^2^**	**D150**	**D300**	**D450**
C14:0	6.83 ± 2.75^B^	10.25 ± 5.85^B^	21.80 ± 12.26^A^
C16:0	399.96 ± 123.05^b^	458.28 ± 170.47^b^	764.19 ± 374.79^a^
C18:0	198.99 ± 52.20^b^	208.20 ± 64.40^b^	308.56 ± 150.86^a^
Others	14.01 ± 2.06^b^	13.64 ± 2.41^b^	18.28 ± 5.30^a^
Total SFA	619.79 ± 178.79^b^	690.37 ± 242.71^b^	1,112.84 ± 540.66^a^
C14:1	3.85 ± 4.81	2.45 ± 0.66	3.05 ± 0.97
C16:1	39.95 ± 19.43^b^	38.82 ± 22.66^b^	78.48 ± 47.78^a^
C18:1n9c	589.09 ± 273.91^b^	655.65 ± 320.59^b^	1,190.42 ± 751.17^a^
Others	14.59 ± 7.34	12.46 ± 3.48	17.05 ± 6.87
Total MUFA	647.47 ± 299.95^b^	709.38 ± 346.47^b^	1,289 ± 802.87^a^
C18:2n6c	265.78 ± 114.97^B^	289.78 ± 114.09^B^	588.79 ± 284.92^A^
C18:3n6	2.32 ± 0.62^B^	2.99 ± 1.25^B^	6.99 ± 4.05^A^
C20:3n6	11.35 ± 2.75	10.75 ± 2.36	13.18 ± 4.11
C20:4n6	182.77 ± 21.92	179.60 ± 19.21	189.69 ± 40.14
Total n-6 PUFA	462.22 ± 116.78^B^	483.12 ± 126.74^B^	798.65 ± 316.44^A^
C18:3n3	6.57 ± 3.45^B^	8.90 ± 4.92^B^	19.63 ± 10.01^A^
C20:3n3	1.55 ± 0.17^B^	1.92 ± 0.16^A^	2.07 ± 0.30^A^
C20:5n3	1.83 ± 0.21^A^	1.53 ± 0.10^B^	1.75 ± 0.17^A^
C22:6n3	16.16 ± 2.57	16.17 ± 3.92	20.86 ± 8.92
Total n-3 PUFA	26.10 ± 3.27^B^	28.52 ± 6.22^B^	44.30 ± 14.66^A^
Total PUFA	488.32 ± 119.55^B^	511.64 ± 131.11^B^	842.95 ± 329.11^A^

1 *Values within a row followed by different superscript letters (a–c) differ significantly (p ≤ 0.05) and values within a row followed by different capital superscript letters (A–C) differ significantly (p ≤ 0.01). DM, dry matter*.

2 *SFA, saturated fatty acid; MUFA, monounsaturated fatty acid; PUFA, polyunsaturated fatty acid*.

Indices of FA metabolism (elongase, thioesterase, and desaturase) and related nutritional indices (n-6/n-3 and PUFA/SFA) were summarized in Table 2. The indices of elongase and thioesterase exhibited a gradually decreased trend with the age increasing (*P* < 0.05). However, delta-9 desaturase for C18 (SCD18) showed an increased trend (*P* < 0.05) with the age increasing. The value of SCD18 at D450 was higher (*P* < 0.05) than that at D150 or D300, which did not differ from each other (*P* > 0.05). In addition, the indices of delta-9 desaturase for C16 (SCD16), delta-5 desaturase (D5D), and delta-6 desaturase (D6D) exhibited no significant difference during the laying period (*P* > 0.05). As for the nutritional indices of PUFA/SFA and n-6/n-3, no significant differences (*P* > 0.05) were observed during the laying period.

**Table 2:** Main estimated indices of fatty acid metabolism (on the basis of fatty acid composition expressed as μg/g of DM) in skinless breast meat during laying period (mean ± SD, *n* = 10)^1^.

**Item^2^**	**D150**	**D300**	**D450**
Elongase	0.51 ± 0.05^a^	0.46 ± 0.04^b^	0.41 ± 0.05^c^
Thioesterase	60.93 ± 9.41^a^	50.13 ± 12.88^b^	37.60 ± 7.33^c^
SCD16	0.096 ± 0.019	0.079 ± 0.018	0.097 ± 0.028
SCD18	2.85 ± 0.55^b^	3.01 ± 0.54^b^	3.65 ± 0.82^a^
D5D	16.92 ± 4.06	17.49 ± 4.31	15.24 ± 4.24
D6D	0.009 ± 0.002	0.010 ± 0.002	0.012 ± 0.004
n-6:n-3 PUFA ratio	17.52 ± 2.80	17.06 ± 3.49	17.99 ± 3.59
PUFA:SFA ratio	0.80 ± 0.06	0.76 ± 0.08	0.78 ± 0.09

1 *Values within a row followed by different superscript letters (a–c) differ significantly (p ≤ 0.05). DM, dry matter*.

2 *SCD16, Delta-9 desaturase for C16; SCD18, Delta-9 desaturase for C18; D5D, Delta-5 desaturase; D6D, Delta-6 desaturase*.

### Proteomic Expression Profiling of Breast Muscle

A total of 85,680 match spectra, 41,768 peptides, and 4,935 proteins were identified with an FDR < 1%. Details of all the accurately identified proteins were shown in Supplementary Tables S1, S2. All proteins were grouped according to biological process (BP), cellular component (CC), and molecular function (MF) by GO analysis (Supplementary Table S3). The macromolecule metabolic process, regulation of the cellular process, and cellular macromolecule metabolic process were main categories in BP. The intracellular, intracellular part, and cytoplasm were main categories in CC. The anion binding, cation binding, and nucleotide binding were the three most abundant categories in MF (Supplementary Figure S1). The pathway enrichment analysis indicated that these proteins were primarily involved in the pathway of transport and catabolism, translation, signal transduction, and carbohydrate metabolism (Supplementary Figure S2).

### Identification of Differentially Expressed Proteins

**Figure 1:**
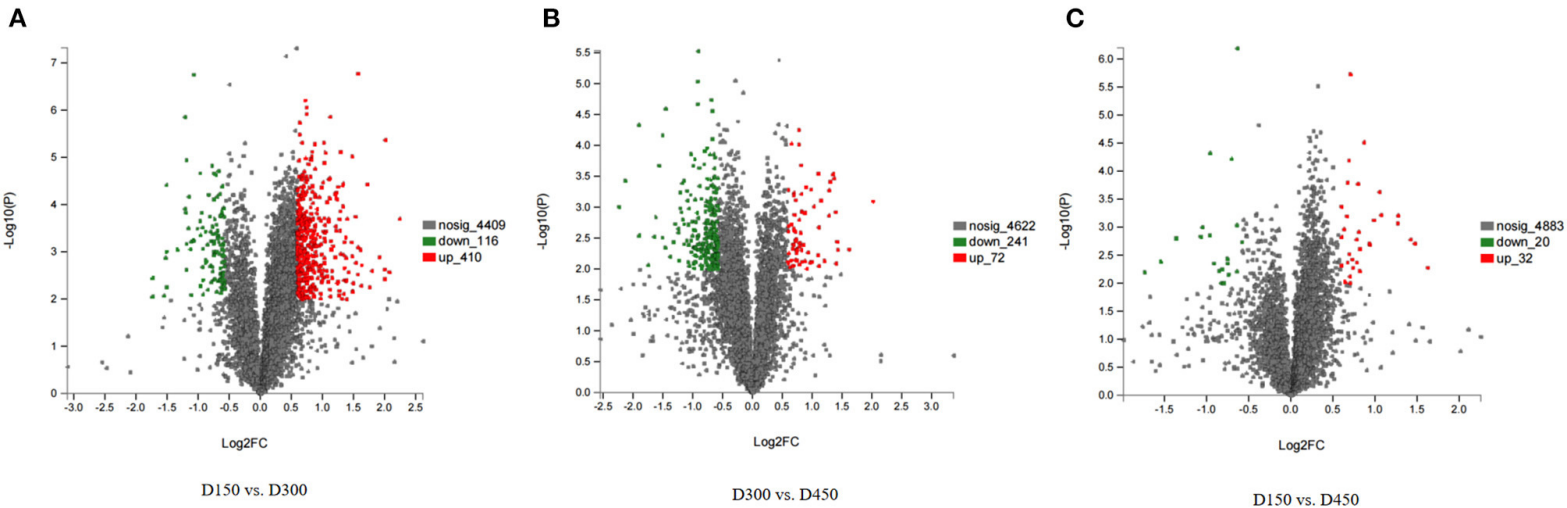
Volcano plots of the univariate statistical analysis results. D150 vs. D300 **(A)**, D300 vs. D450 **(B)**, and D150 vs. D450 **(C)**. The red dots indicate significantly upregulated proteins (*P* < 0.01 and FC > 1.5), and the green dots indicate significantly downregulated proteins (*P* < 0.01 and FC < 0.67). The gray dots represent proteins with non-significant (*P* < 0.01 and 0.67 < FC < 1.5) differences in expression.

Among the total identified proteins, 664 DEPs (FC > 1.50 or < 0.67 and *P* < 0.01) was selected to assess dynamic changes in proteins at the three different chronological ages of BYC hens. In the 664 DEPs There were 410 up- and 116 down-regulated proteins in D150 vs. D300 group, 32 up- and 20 down-regulated proteins in D150 vs. D450 group, and 72 up- and 241 down-regulated proteins in D300 vs. D450 group (Supplementary Table S4). The number of DEPs in the D150 vs. D450 group was lesser than that in the two groups of D150 vs. D300 and D300 vs. D450, indicating that the protein profiles at D300 were greatly different from D150 and D450. Volcano plots were generated to visualize the data for the three pairwise comparison groups (D150 vs. D300, D300 vs. D450, and D150 vs. D450) (Figures 1A–C). The red dots indicated significantly upregulated proteins (*P* < 0.01 and FC > 1.50), while the green dots indicated significantly downregulated proteins (*P* < 0.01 and FC < 0.67). The gray dots represented proteins that showed non-significant (*P* > 0.01 or 0.67 < FC < 1.50) differences in expression. According to the *P-*values, the top 15 DEPs of each comparison group were listed in Table 3. These DEPs could play a crucial role in FA metabolism, development of muscle, and act as candidate genes for further study.

**Table 3:** The top 15 differentially expressed proteins in three comparison groups (D150 vs. D300, D300 vs. D450, and D150 vs. D450).

**Accession number**	**Gene symbol**	**Protein name**	**Fold change**	***P*** **-value**
**D150 vs. D300**				
P16419	MYBPC2	Myosin-binding protein C, fast-type	0.475	0.00000
F1NK46	SPOUT1	Uncharacterized protein	2.965	0.00000
F1NHL0	CFAP57	Cilia and flagella associated protein 57	0.430	0.00000
E1BTT4	N/A	Hydroxyacyl-CoA dehydrogenase trifunctional multienzyme complex subunit beta	1.645	0.00000
E1C697	GPX7	Glutathione peroxidase	1.667	0.00000
A0A1L1S0D8	PTBP1	Uncharacterized protein	1.668	0.00000
P00337	LDHB	L-lactate dehydrogenase B chain	2.176	0.00000
A0A3Q3AXQ2	LOC107057318	Fat storage inducing transmembrane protein 2	1.550	0.00000
A0A1D5PGW7	SRI	Uncharacterized protein	1.547	0.00000
A0A1D5PCL3	MYBPC1	Myosin binding protein C, slow type	4.023	0.00000
P53488	ACTR2	Actin-related protein 2	1.589	0.00001
Q98UJ8	N/A	2-oxoisovalerate dehydrogenase subunit alpha (Fragment)	1.832	0.00001
F1NFA8	NUP62	Nucleoporin 62	2.028	0.00001
P02272	H2AZ2	Histone H2A.V	1.774	0.00001
F1NI29	HADHA	Uncharacterized protein	2.443	0.00001
**D150 vs. D450**				
E1C897	CTSEAL	CathepsinE-A-like protein	0.644	0.00000
O57348	N/A	Cellular nucleic acid binding protein	1.629	0.00000
A0A1D5P5L5	LOC107048987	Methyltransf_11 domain-containing protein	1.821	0.00003
P02752	N/A	Riboflavin-binding protein	0.512	0.00005
F1NVP2	FGD6	FYVE, RhoGEF, and PH domain containing 6	0.612	0.00006
A0A3Q2UI73	N/A	Uncharacterized protein	1.605	0.00006
A0A3Q2TXD8	N/A	Uncharacterized protein	1.591	0.00016
Q6WEB3	N/A	Thymosin beta	1.737	0.00017
F1NPN5	SPIA3	SERPIN domain-containing protein	2.071	0.00023
P18302	DBN1	Drebrin	1.514	0.00043
E1C206	SPIA5	SERPIN domain-containing protein	2.099	0.00060
P08250	apoA-I	Apolipoprotein A-I	2.416	0.00062
A0A1D5PDE6	MARCKS	Myristoylated alanine-rich C-kinase substrate	1.582	0.00064
Q0GFE9	TMSB15B	Thymosin beta	1.980	0.00073
B3TZB6	N/A	PNPLA1 (Fragment)	2.399	0.00086
**D300 vs. D450**				
E1C697	GPX7	Glutathione peroxidase	0.531	0.00000
F1NJ53	SMPX	Uncharacterized protein	0.527	0.00001
F1NSW0	VAPB	MSP domain-containing protein	0.620	0.00002
A0A1D5NT61	GLUD2	ELFV_dehydrog domain-containing protein	0.527	0.00002
A0A3Q2UE06	LOC100857512	Protein kinase domain-containing protein	0.364	0.00003
F1P174	MIPEP	Mitochondrial intermediate peptidase	0.626	0.00003
A0A1D5PFK7	AP4E1	Adaptor related protein complex 4 subunit epsilon 1	0.267	0.00005
F1NCA2	GPD2	Glycerol-3-phosphate dehydrogenase	1.709	0.00006
A0A3Q2UDY7	INO80	Uncharacterized protein	0.351	0.00007
A0A3Q3A5U5	N/A	Mitochondrial ribosomal protein S24	0.628	0.00008
A0A1D5PRC4	ZNF236	Zinc finger protein 236	1.562	0.00009
F1NVK5	MIER2	MIER family member 2	1.710	0.00010
F1P310	COQ9	Ubiquinone biosynthesis protein	0.587	0.00011
A0A1D5PKI5	GMDS	GDP-mannose 4,6-dehydratase	0.572	0.00012
Q76MS9	MUSTN1	Musculoskeletal embryonic nuclear protein 1	0.487	0.00014

**Figure 2:**
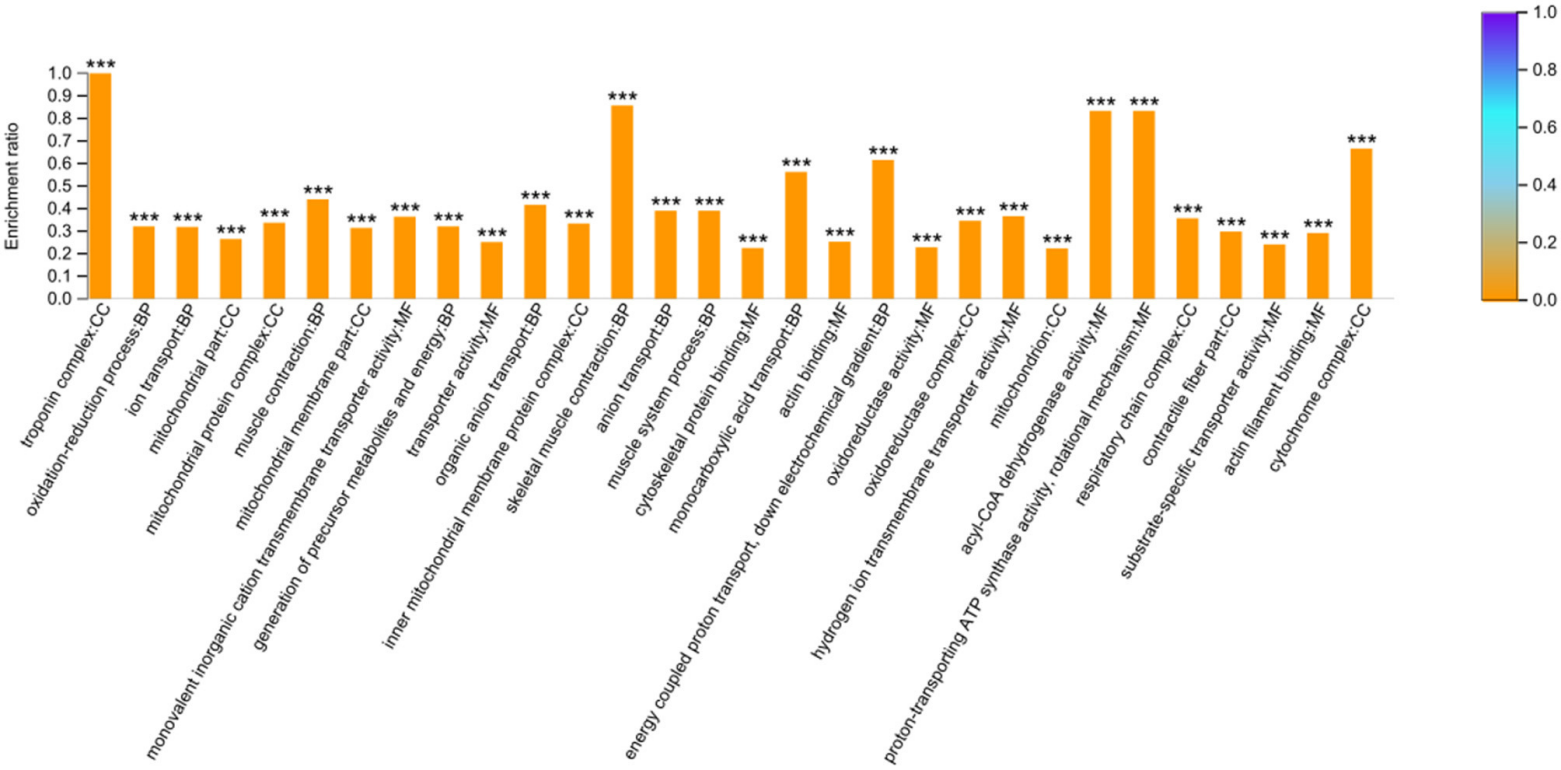
GO enrichment analysis based on the 664 differentially expressed proteins. The top 10 categories in biological process (BP), cellular component (CC) and molecular function (MF) were presented. The ^***^ symbol indicates the *P*-values <0.001.

To gain insights into the functions of these 664 DEPs, GO and KEGG enrichment analyses were carried out. The analysis showed that 344 GO terms were enriched (*P* < 0.05) and the top 10 categories in BP, CC, and MF were shown in Figure 2. The DEPs in the BP group were mainly involved in the oxidation-reduction, ion transport, and muscle contraction. The DEPs in the CC group were primarily enriched in the troponin complex, mitochondrial part, and mitochondrial protein complex. And the DEPs in the MF group were mainly distributed in monovalent inorganic cation transmembrane transporter activity, transporter activity, and cytoskeletal protein binding (Supplementary Table S5).

**Figure 3:**
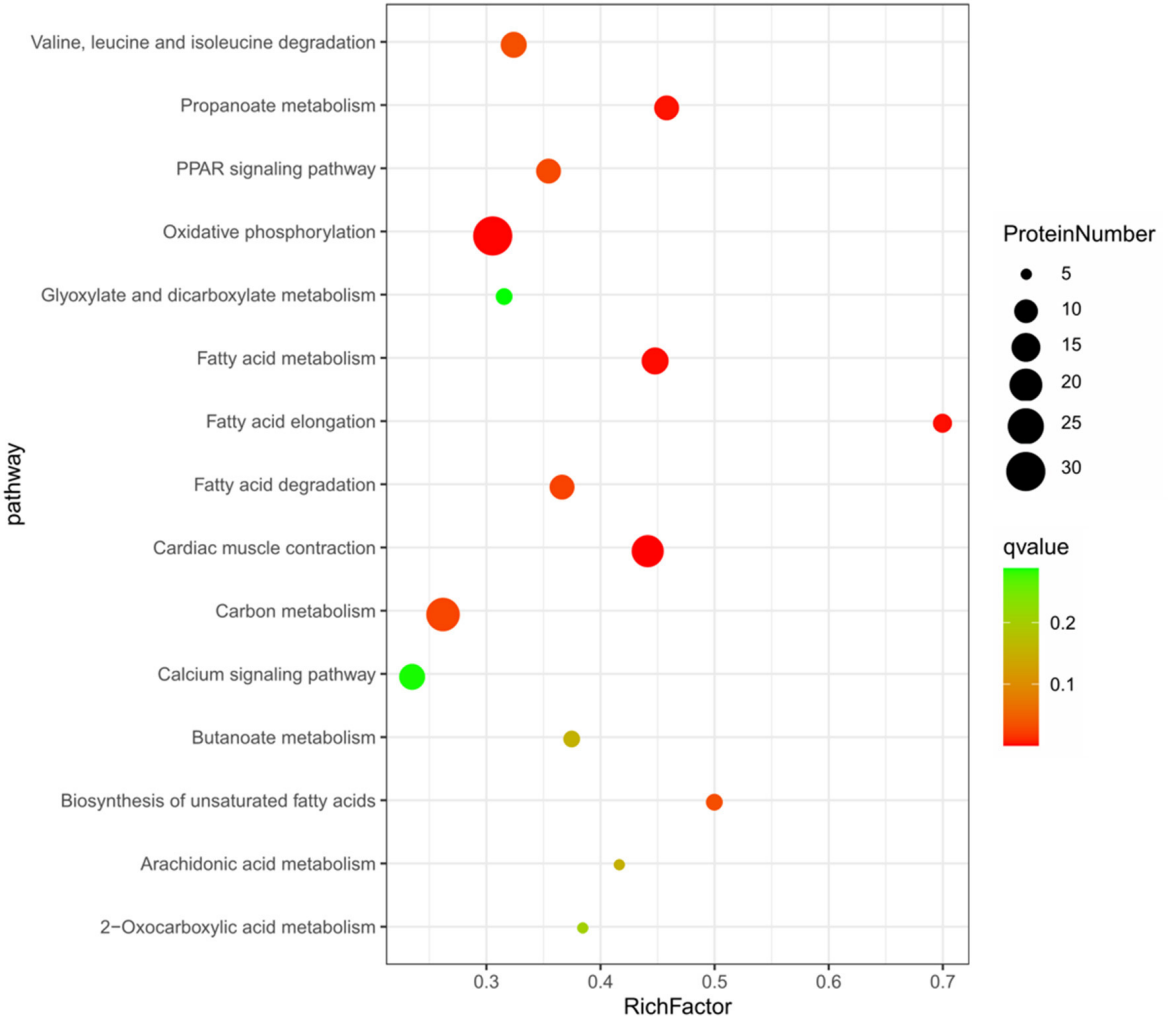
The top 15 enriched KEGG pathway based on the 664 differentially expressed proteins.

The KEGG pathway enrichment analysis of the 664 DEPs (Figure 3) showed that 15 pathways were significantly enriched (*P* < 0.05), including some well-known pathways affecting FA synthesis (FA metabolism, FA elongation, FA degradation, biosynthesis of unsaturated FAs pathways, and arachidonic acid metabolism), signaling pathways (PPAR and calcium), and the pathways related to muscle contraction and metabolism of energy or amino acid (cardiac muscle contraction, oxidative phosphorylation, propanoate metabolism, carbon metabolism, butanoate metabolism, 2-oxocarboxylic acid metabolism, glyoxylate and dicarboxylate metabolism, as well as valine, leucine and isoleucine degradation pathways). About half of these pathways were involved in FA synthesis and signaling pathways related to lipid metabolism (Supplementary Table S5).

### The DEPs Related to Fatty Acid or Lipid Metabolism

**Figure 4:**
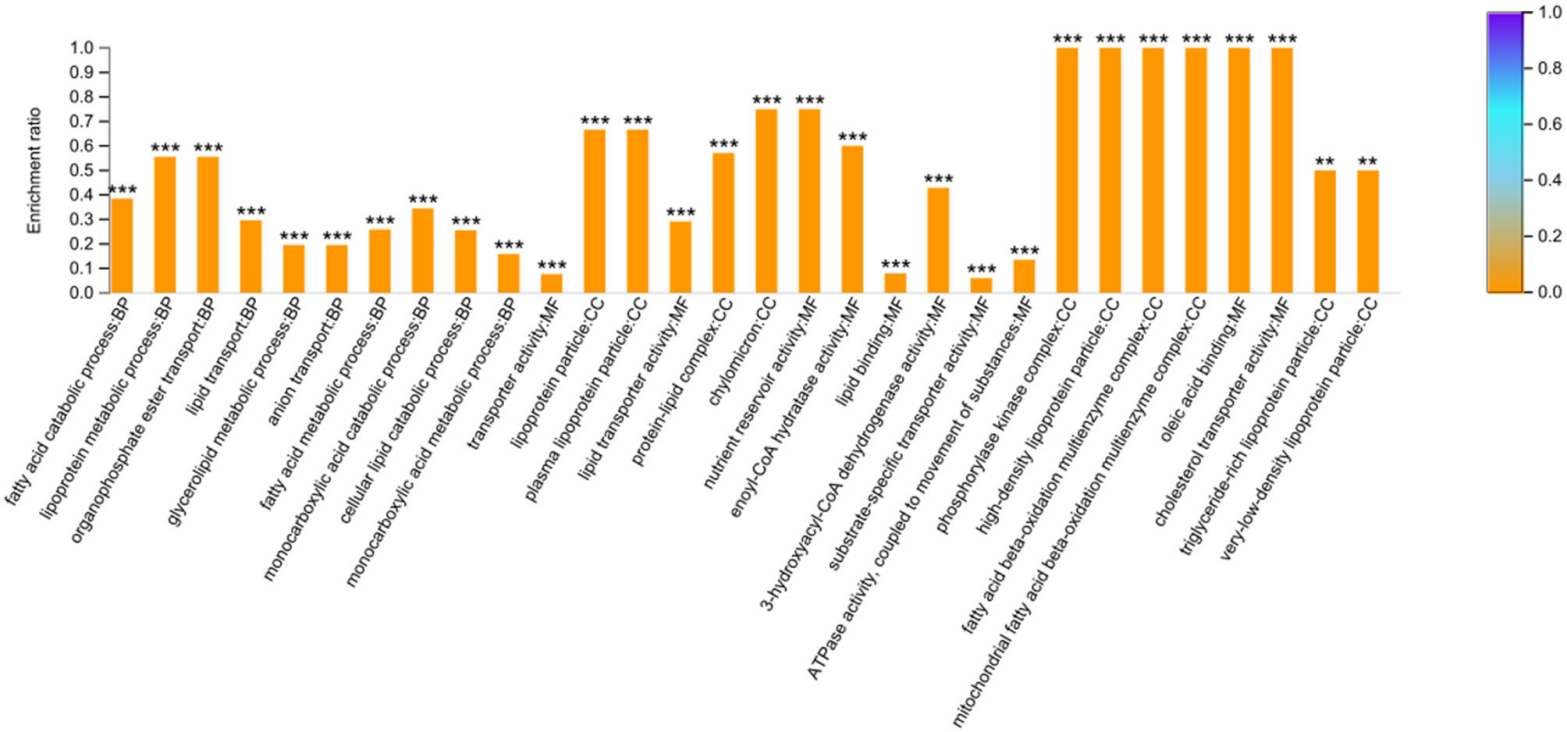
GO enrichment analysis based on the 57 differentially expressed proteins related to fatty acid metabolism. The top 10 categories in biological process (BP), cellular component (CC) and molecular function (MF) were presented. The symbol ^***^ and ^**^ indicates the *P*-values <0.001 and *P*-values < 0.01, respectively.

A total of 34 DEPs related to the FA or lipid metabolism were screened according to the enriched GO terms (Supplementary Table S6). These 34 DEPs were mainly involved in the FA metabolic process (such as ACAT1, ECHDC1, CPT2, DECR1, ACADS, ABCD2, HADHA, ACOT11, ABHD2, ACADL, HADH, ACACA, CPT1A, and TECRL), and FA/lipid transport (such as ANXA1, ABCC4, FABP, FABP3, ATP8B4, apoC-III, apoA-IV, apoA-I, apoB, VTG1, and VTG2). On the other hand, a total of 37 DEPs were screened based on the KEGG enrichment pathways related to FA metabolism and some well-known signaling pathways related to lipid metabolism (PPAR, MAPK, Calcium, TGF-beta, and Wnt). To comprehensively understand regulatory mechanism of FA-related metabolism based on proteomic analysis, the DEPs related to FA or lipid metabolism according to the enrichment results of GO terms and KEGG were combined for further analysis. There were 14 overlapping DEPs in both GO terms and KEGG. Finally, 57 DEPs were successfully obtained (Supplementary Table S6), which were enriched into 562 GO terms (*P* < 0.05). The top 10 categories in BP, CC, and MF were shown in Figure 4. The DEPs in the BP group were mainly distributed in the FA catabolic process, lipoprotein metabolic process, and organophosphate ester transport, etc. The DEPs in the CC group were enriched primarily on the lipoprotein particle, plasma lipoprotein particle, and protein-lipid complex, etc. And the DEPs in the MF group were primarily distributed in the transporter activity, lipid transporter activity, and nutrient reservoir activity, etc. (Supplementary Table S7).

**Figure 5:**
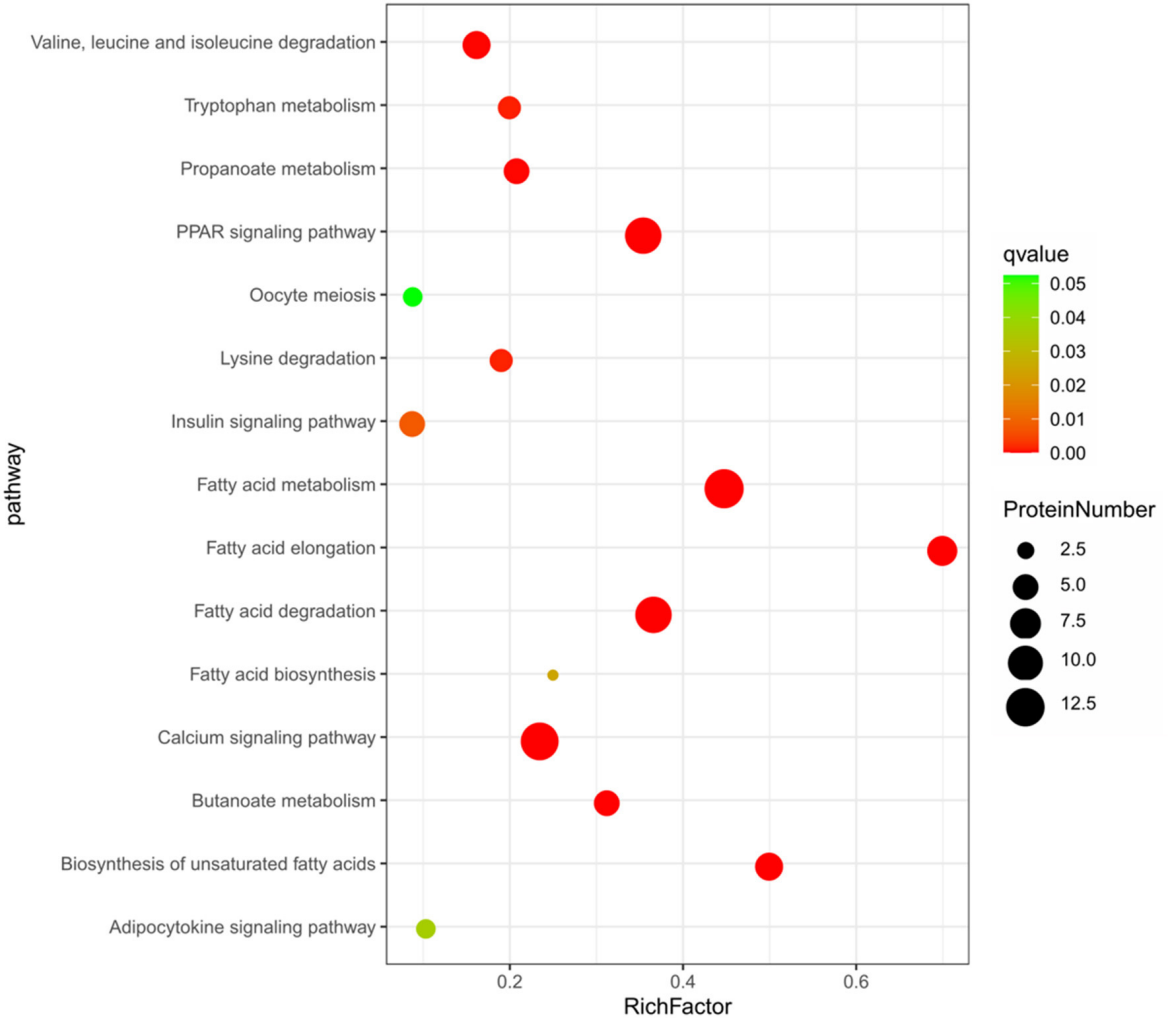
The top 15 enriched KEGG pathway based on the 57 differentially expressed proteins.

The KEGG analysis showed that a total of 21 pathways were significantly enriched (*P* < 0.05). The top 15 enriched pathways were shown in Figure 5, including some well-known pathways affecting FA synthesis (FA metabolism, FA elongation, FA degradation, and biosynthesis of unsaturated FA), the signaling pathways related to lipid metabolism (PPAR, calcium, insulin, adipocytokine, and VEGF), and other pathways related to amino acid metabolism (lysine degradation, tryptophan metabolism, beta-alanine metabolism, as well as valine, leucine and isoleucine degradation). More than half of these pathways were involved in FA synthesis and signaling pathways related to lipid metabolism as well (Supplementary Table S7).

### Expression Patterns of DEPs Related to Fatty Acid or Lipid Metabolism

**Figure 6:**
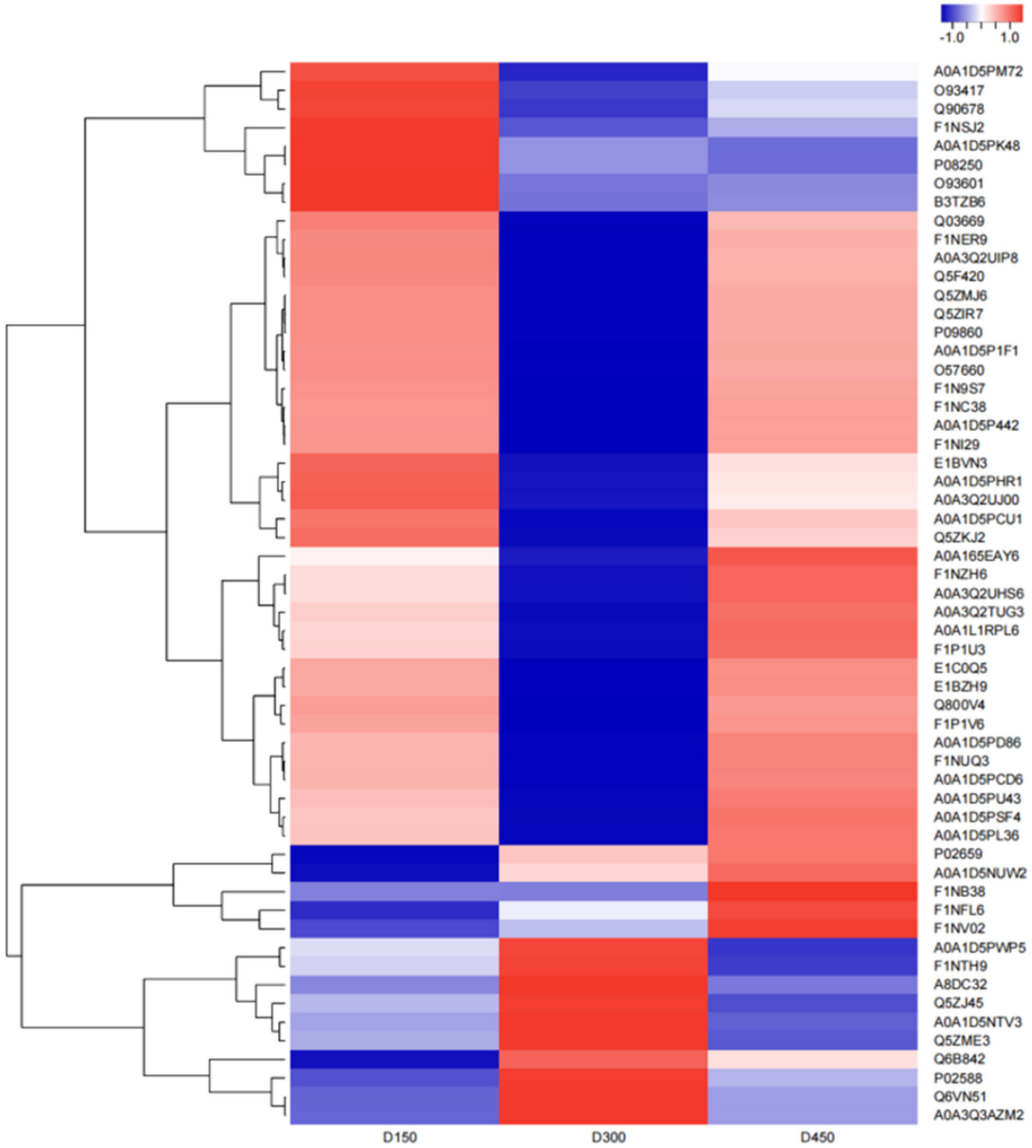
Heatmap of the 57 differentially expressed proteins related to fatty acid metabolism in the three different chronological ages (D150, D300, and D450) of breast muscle. Blue indicates downregulation, red indicates upregulation, and white indicates undetectable in the heat map.

**Figure 7:**
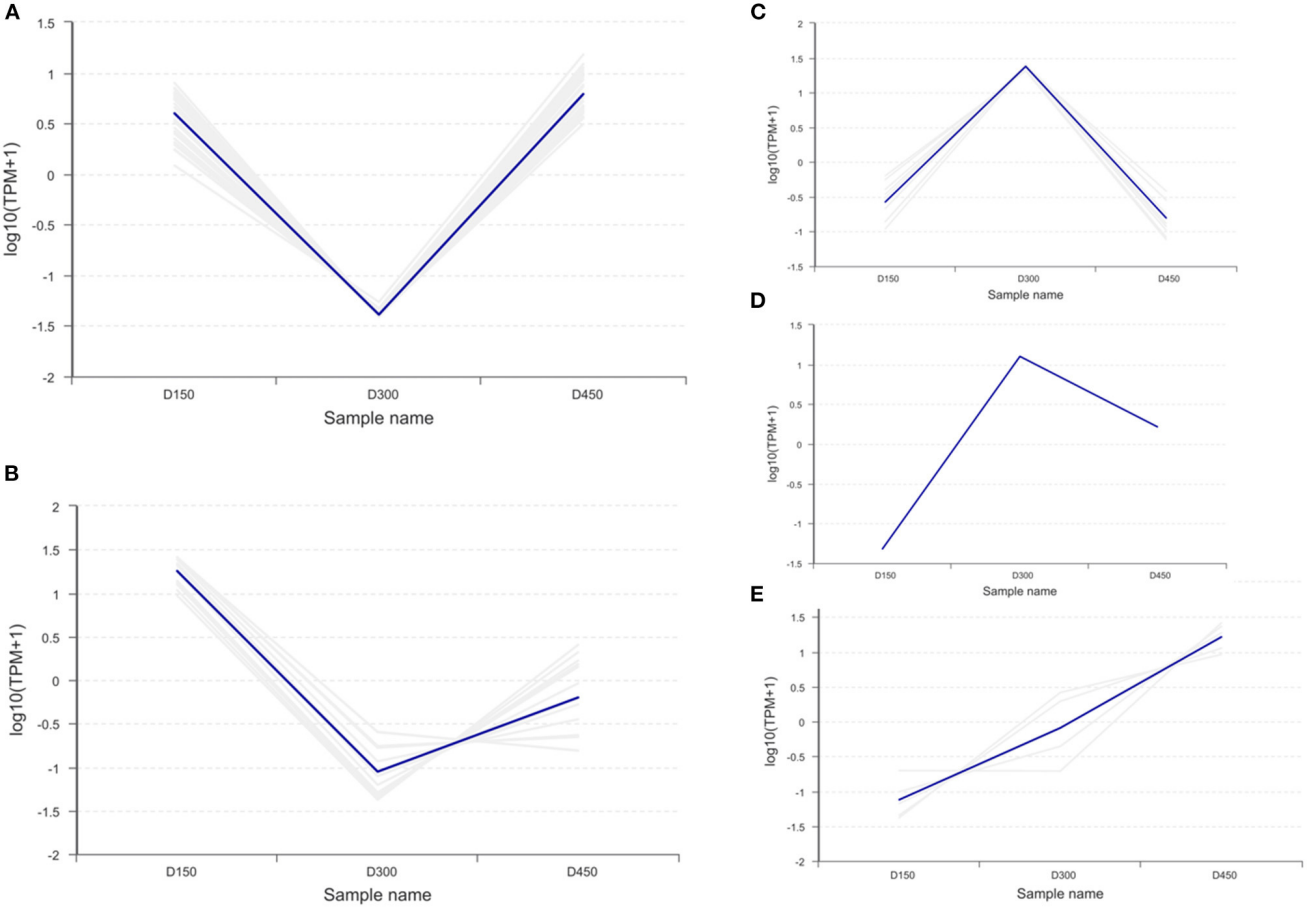
Clustering analysis of the 57 differentially expressed proteins related to fatty acid metabolism in the three different chronological ages (D150, D300, and D450) of breast muscle. **(A)** Cluster 1 (29 proteins) down-regulated significantly from D150 to D300, then up-regulated sharply from D300 to D450. **(B)** Cluster 2 (13 proteins) down-regulated from D150 to D300 then up-regulated slowly from D300 to D450. **(C)** Cluster 3 (9 proteins) up-regulated steadily from D150 to D300 then sharply down-regulated from D300 to D450. **(D)** Cluster 4 (1 proteins) up-regulated sharply from D150 to D300, then slowly down-regulated from D300 to D450. **(E)** Cluster 5 (5 proteins) up-regulated consistently from D150 to D450.

The hierarchical cluster analysis was used to compare the differences in the expression patterns of 57 DEPs between the three chronological ages. The results are shown as a heatmap (Figure 6). The expression patterns of the DEPs at D150 and D450 were similar to each other compared with those at D300 by the clustering analysis. Based on the expression pattern, five distinct clusters could be established. The largest cluster (cluster 1, including 29 proteins) was down-regulated significantly from D150 to D300, and then up-regulated sharply from D300 to D450. However, cluster 2 (13 proteins) showed more sharply down-regulated from D150 to D300 and then up-regulated slowly from D300 to D450. However, the contents were still lower than those at D150. On the contrary, the nine proteins of cluster 3 were up-regulated from D150 to D300, and then sharply down-regulated from D300 to D450. It was worth noting that cluster 4 contained only one protein, which sharply up-regulated from D150 to D300 but showed much slower down-regulated from D300 to D450. In addition, cluster 5 (5 proteins) had a consistent overall pattern of up-regulation from D150 to D450 (Figure 7).

### Interaction Network of DEPs Related to Fatty Acid or Lipid Metabolism

**Figure 8:**
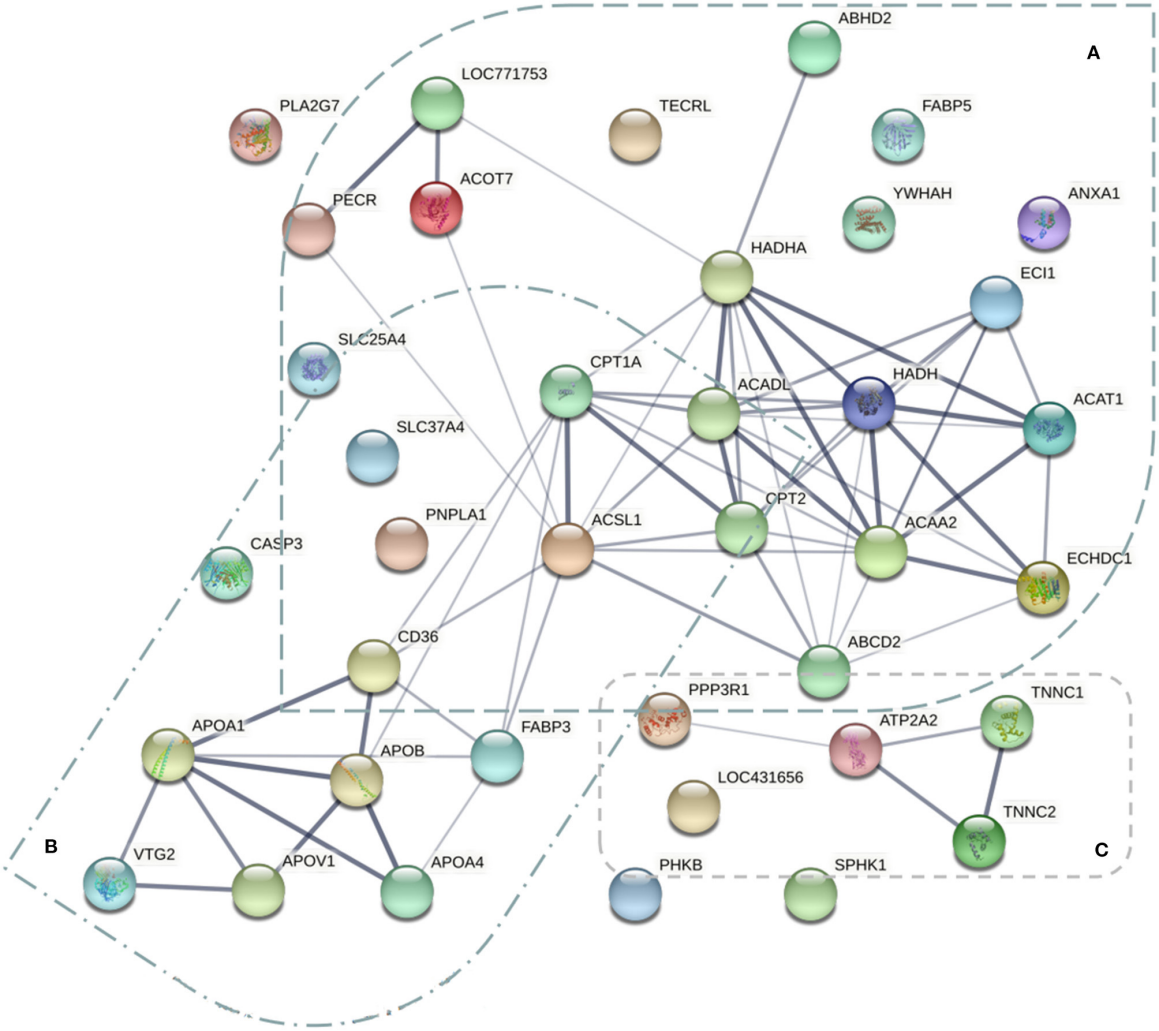
The protein-protein interaction network of the 57 differentially expressed proteins related to fatty acid metabolism of breast muscle. Three functional modules were apparent in the network, forming tightly connected clusters. **(A)** The proteins related to pathways of fatty acid metabolism. **(B)** The proteins involved the lipid transport. **(C)** The proteins associated with calcium signaling pathway.

To explore the protein interaction networks related to chicken breast meat FA and lipid metabolism across the laying period, the protein-protein interaction network of the DEPs identified in this study was also analyzed using web-tool STRING 11.0 (http://string-db.org). There were three functional modules (Figure 8). The first was related to pathways of FA synthesis, including FA metabolism (PECR, HADHA, CPT1A, ACADL, HADH, ACAT1, ACSL1, CPT2, and ACAA2), FA elongation (LOC771753, ACOT7, HADHA, HADH, and ACAA2), FA degradation (HADHA, ECI1, CPT1A, ACADL, HADH, ACAT1, ACSL1, CPT2, and ACAA2) and biosynthesis of unsaturated FAs (LOC771753, PECR, and ACOT7). This module might be related to the significant FA changes in breast muscle during the laying period. The second module involved lipid transport (VTG2, apoA-I, apoB, apoA-IV, and FABP3), which might account for the transportation of FA. The third module was associated with the calcium signaling pathway (PPP3R1, ATP2A2, TNNC1, and TNNC2).

### Validation of the TMT-Based Results by PRM

**Figure 9:**
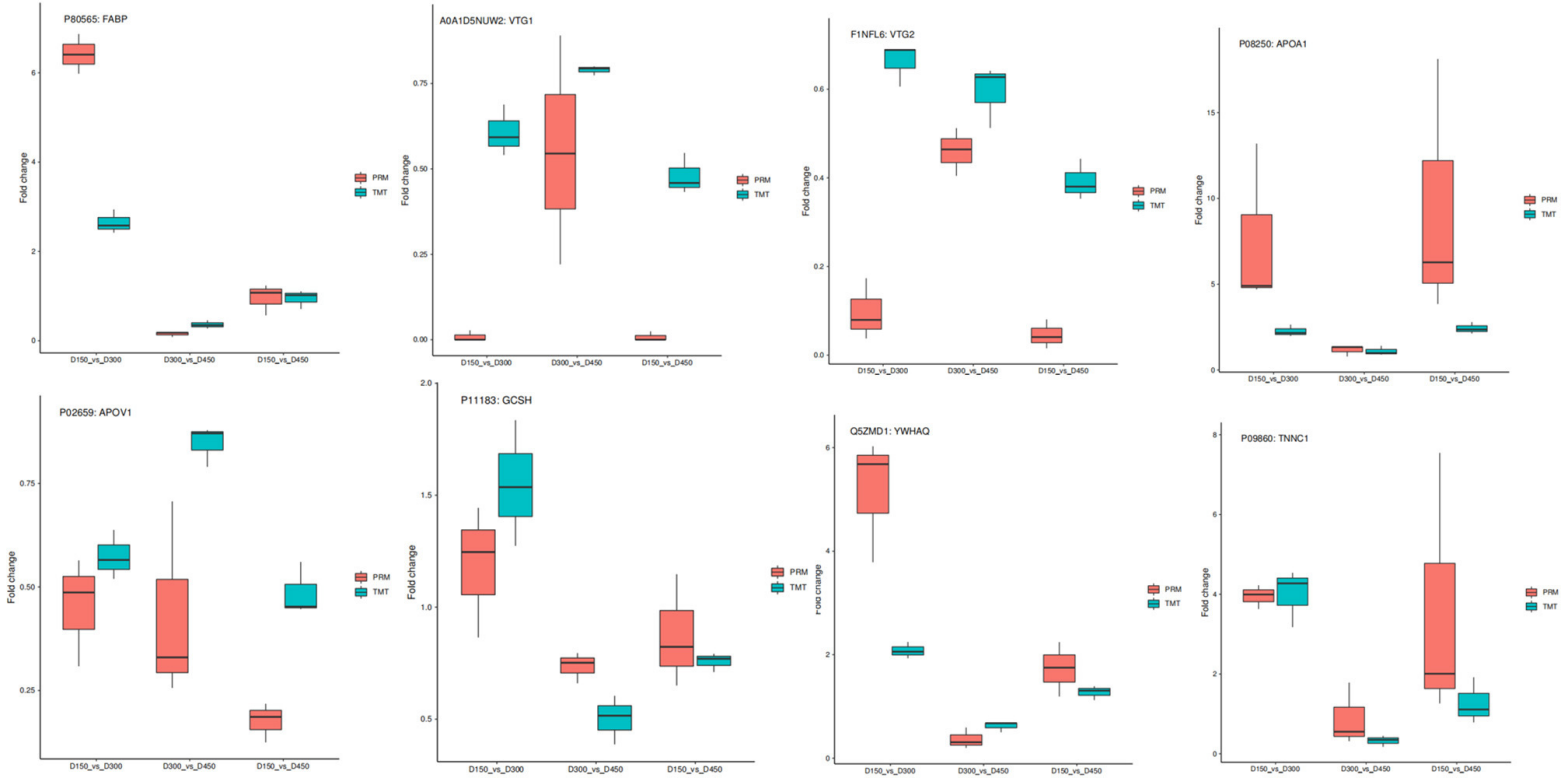
Relative expression levels of selected proteins measured by PRM and TMT in the three comparison groups of D150 vs. D300, D300 vs. D450, and D150 vs. D450.

To assess the validity of the TMT data, a total of eight target function proteins, including five proteins (FABP, VTG1, VTG2, apoA-I, and apoA-IV) related to FA transportation and three proteins (GCSH, YWHAQ, and TNNC1), related to glycine cleavage system, identical protein binding, and calcium signaling pathway, respectively, were selected for verification using PRM quantitative analysis at the three different chronological ages. As shown in Figure 9, overall, the PRM results exhibited similar trends with the TMT data, supporting the plausibility and reliability of the proteomics data.

## Discussion

The objective of this study was to investigate the effect of hen ages on FA profiles and to provide insight into the regulatory mechanism of FA-related metabolism based on proteomic analysis in breast meat of a slow-growing BYC harvested at 150, 300, and 450 days of laying. To our knowledge, this study, for the first time, examines the effect of hen age on FA in breast meat during the laying period from sexual maturation to culling stage using a high-throughput-proteomic-based approach.

### The Profiles and Related Indices of FA

It has been demonstrated that the age of birds had a profound effect on FA composition in breast meat. The long-chain PUFA and total PUFA levels increased as the age increased (17). In our study, the large differences in the FA composition in the breast meat across the three chronological ages may be attributed chiefly to the chronological age, since all the birds received the same diet. The concentrations of total n-3 PUFA, n-6 PUFA, MUFA, and SFA of BYC increased with age. The concentrations of total n-3 and n-6 PUFA dramatically increased by ~70 and 73% from D300 to D450, whereas they only increased by about 9 and 5% from D150 to D300, respectively. Similarly, the concentrations of MUFA and SFA increased by about 99 and 80%, respectively, from D300 to D450, whereas they just increased by about 10 and 11%, respectively, from D150 to D300 (Table 1). Taken together, the concentrations of FAs showed a significantly rising trend with age increasing, especially during the period from 300 to 450 days. Thus, This result suggested that breast meat of BYC at D450 showed a more desirable nutritional value in the term of the FA concentrations.

The ratios of PUFA/SFA and n-6/n-3 were often used to evaluate the health impact of fat in meat (36) and the recommendations of the ratios were more than 0.4 for PUFA/SFA and <4.0 for n-6/n-3 (37, 38). In our study, the ratios of PUFA/SFA and n-6/n-3 varied in the range of 0.76–0.80 and 17.06–17.99, respectively, and were much greater than the recommended values. These may be due exclusively to the higher levels of C18:2n-6 and C20:4n-6, the major n-6 PUFAs that contributes to total PUFA in breast meat based on the corn meal diet, as the diet affected the FA profile (39, 40). Furthermore, we found no differences among the three laying ages regarding the ratios of PUFA/SFA and n-6/n-3, which illustrated that the laying age did not affect the health impact of fat in breast meat of BYC. These results were consistent with the data published in the previous study (41). However, they disagrees with the findings by Popova et al. (20), who reported that the n-6/n-3 ratio exhibited a decreasing trend from age 9 to 18 weeks. The genotype, diet, and age of the bird might be the reasons for the difference.

The indices SCD16, SCD18, D5D, and D6D have been used to estimate the enzyme activity in the synthesis of MUFA and PUFA (11, 34, 42). These 4 indices above are the estimators for the ability of enzymes to insert double bonds in essential FA to obtain long-chain FA (34). In the present study, only the SCD18 index tended to increase as the hens became older; however, no differences were detected for the other three indices (Table 2). This could be attributed to the higher substrate MUFA for the SCD18 in the breast meat of the older laying hens. Moreover, in FA synthesis, elongase and thioesterase were responsible for the synthesis and termination or release of the newly synthesized FA (43). The ratios of C16:0 to C14:0 and C18:0 to C16:0 are used to calculate the indices of thioesterase and elongase, respectively (33). In this study, our data indicated that elongase and thioesterase decreased as the age of birds increased (Table 2). Hence, the lower thioesterase and elongase indices observed in older birds resulted from reduced cleavage of C16-acyl-acyl carrier protein and efficiency in incorporation from C16:0 to C18:0.

### The Proteins Participated in the Regulation of FA Metabolism

FA metabolisms are a dynamic process regulated comprehensively by enzyme activities, hormones, and cell factors, including a series of steps such as desaturation, elongation, and β-oxidation (2). In this study, KEGG pathway analysis showed a total of 16 DEPs (ACACA, ACAA2, ACOT4, ACOT7, ECI1, ACADS, ACAT1, ACADL, ACSL1, HADH, HADHA, HSD17B12, CPT2, CPT1A, PECR, CFR-associated protein p70) among these screened 57 DEPs were significantly enriched in the pathways of FA metabolism (gga01212), degradation (gga00071) and elongation (gga00062). For the three pathways mentioned above, four common DEPs (HADH, HADHA, ACAA2, and CFR-associated protein p70) were found, indicating that there are interactions between these pathways. These results were further confirmed by the protein-protein interaction network data (Figure 8), suggesting that HADH, ACAA2, HADHA, and CFR-associated protein p70 (O57660) played a critical role in the metabolism of FA. This finding is consistent with the findings of the previous studies (26).

Of these 16 DEPs involved in the FA pathways of metabolism (gga01212), degradation (gga00071), and elongation (gga00062), the expression of 14 DEPs (ACAA2, ACOT4, ACOT7, ECI1, ACADS, ACAT1, ACADL, ACSL1, HADH, HADHA, HSD17B12, CPT2, PECR, and CFR-associated protein p70) were significantly reduced but the expression of ACACA and CPT1A were increased at D300 than at D150 and D450 (Supplementary Table S4; Figure 7). It was noteworthy that the older birds exhibited reduced indices of elongase and thioesterase but increased indice of SCD18 (Table 2). These results indicated that laying age played a crucial role in the pathways of FA metabolism, degradation, and elongation.

### The Proteins Related to Lipid and FA Transport

The chicken *de novo* synthesis of FA mainly occurs in the liver (44–46), and then exported to other tissues in the form of triglyceride-rich very low-density lipoproteins (VLDL) through the peripheral vascular system (47, 48). In the present study, GO-term analysis revealed that there were 8 DEPs (FABP, FABP3, ATP8B4, apoA-I, apoC-III, apoA-IV, ANXA1, and ABCC4) and 14 DEPs (ATP8B4, ATP2A3, ATP2A2, SLC25A4, SLC25A5, SLC37A4, apoA-I, apoA-IV, apoB, ABCC4, ABCD2, VTG1, VTG2, and MTP) involved in lipid transportation (GO:0006869) and transporter activity (GO:0005319), respectively. Among these DEPs, FA-binding proteins (FABPs) are not only considered as a FA transporter from cell membrane to the intracellular sites of FA utilization (49), but also serve as a reservoir for intracellular non-esterified FA (NEFA) and fatty acyl-CoA thioesters (2). The laying age from D150 to D300 exhibited a negative effect on the expression abundances of the FABP and FABP3. However, the laying age from D300 to D450 showed a positive impact on the level of the proteins. Teltathum and Mekchay (50) has shown that the age of Thai indigenous chicken had an antagonistic relationship at 0, 3, 6, and 18 weeks of age. These results demonstrate that the laying age affects the expression of FABPs.

Previous studies have indicated that the principal function of apolipoproteins is working as the vehicle for lipid transport in the intravascular and extravascular compartments (51, 52). Among these apolipoproteins, apoA-I, apoA-IV, and apoC-III are the important components of high-density lipoprotein (HDL). Furthermore, it is believed that apoA-I and apoA-IV activate lecithin:cholesterol acyltransferase (LCAT), and apoC-III inhibits the activation of lipoprotein lipase (LPL) by apoC-II (53, 54). On the other hand, the major apolipoprotein in low density lipoproteins (LDL) is apoB in our analysis. The expression of the apoB increased with age increasing; however, apoA-I, apoA-IV, and apoC-III, which were classified into clusters 2, showed the lowest value at D300 (Supplementary Table S4; Figure 7). These results suggest that the laying age might have different effects on apolipoproteins in HDL and those in LDL.

Interestingly, we also found several VTGs that are related to the yolk protein precursors. The VTGs, including vitellogenin 1 (VTG1) and vitellogenin 2 (VTG2), have been supposedly associated with reproduction and development under estrogen induction (55). Vitellogenin (from Latin *vitellus*, yolk, and gener, to produce) was first named by Pan et al. (56) to describe the female-specific hemolymph protein precursor of egg yolk in insects. VTGs are multidomain proteins and belong to a large family of lipid transfer genes, which play a prominent role in the transportation of the FA to the ovary (57). Furthermore, we found that the expression of the VTGs increased with the age increasing and belonged to cluster 5 (Supplementary Table S4; Figure 7). Our result suggested that the changes in VTGs in breast meat of hens may be involved in balance in fatty acid supplies between meat and yolk during the different ages of laying, since the initiation and maintenance of egg production is an energy-intensive process and requires a large quality of lipids for egg yolk (58). Further research is needed to explore the relationship between the reproduction process (egg yield) and FA compositions of breast meat, which might help to understand the yolk formation *via* VTGs from breast FA.

### The Signaling Pathways Participating in the Regulation of FA Transport and Deposition

It has been suggested that the peroxisome proliferator-activated receptors (PPAR) signaling pathway is involved in regulating adipocyte development and capacity for accumulation of IMF (27, 59–61). Besides, IMF is rich in PUFA composition and content (10). In the present study, the PPAR signaling pathway was detected as well as the other signaling pathways, including calcium, VEGF, and adipocytokine. The DEPs screening showed that 11, 3, 12, and 2 DEPs were involved in signaling pathways of PPAR (gga03320), adipocytokine (gga04920), calcium (gga04020), and VEGF (gga04370), respectively.

CD36 is a membrane receptor that facilitates long-chain FA uptake and enables the inward transport of FA (62–65). In the broiler, the overexpression of CPT1A enhances FA oxidation in hepatocytes and muscle cells and decreases lipid accumulation in chicken (48). Besides, the PPAR signaling pathway plays a transport role in lipid metabolism (57). In the present study, for the signaling pathways of PPAR and adipocytokine, three common DEPs (ACSL1, CD36, and CPT1A) were found, revealing that there might be interactions between two pathways. These results were further confirmed by the protein-protein interaction network data (Figure 8), suggesting that ACSL1, CD36, and CPT1A play a critical role in the transportation and accumulation of FAs. The results are consistent with the findings of the previous studies (48, 66). In addition, from D150 to D300, the laying age exhibited a negative effect on the expression abundances of the CD36 and ACSL1. However, from D300 to D450, the laying age showed a positive impact on the level of the proteins. However, CPT1A presented the opposite trend (Supplementary Table S4; Figure 7). It deduced that FA oxidation was more active and transportation was less active at D300 than those at either D150 or D450.

On the other hand, 12 DEPs were screened according to the calcium signaling pathway. The data of proteomic profiles showed that 5 DEPs (ATP2A2, ATP2A3, SLC25A4, SPHK1, and TNNC1), 1 DEP (SLC25A5), and 6 DEPs (PPP3R1, TNNC2, PHKB, PHKA2, CALM1, and RCJMB04_20n15) belonged to expression patterns of clusters of 1, 2 and 3, respectively (Supplementary Table S4; Figure 7). For the signaling pathways of calcium and VEGF, two common DEPs (PPP3R1 and SPHK1) were found, revealing that there were the interactions between these two pathways. These results were further confirmed by the protein-protein interaction network data (Figure 8), suggesting that PPP3R1 and SPHK1 played a critical role on FA deposition. The results are consistent with the findings of previous studies (67, 68). Additionally, it was noteworthy that the PPP3R1 was enriched in signaling pathways of the calcium (gga04020), VEGF (gga04370), MAPK (gga04010), and Wnt (gga04310) simultaneously.

In brief, this study investigated the effect of laying ages on FA profiles in breast meat of hens and evaluated the TMT-LC-MS/MS proteomics for quantitative analysis of corresponding changes in FA/lipid-related metabolisms. A total of 664 DEPs were identified at the three different laying stages from sexual maturation stage to culling stage. Our data demonstrated that laying age can affect not only FA compositions but also the related metabolisms in breast meat, and provided a novel insight into FA composition discrepancy and mechanism in chicken breast meat during the laying stage.

## Data Availability Statement

The datasets presented in this study can be found in online repositories. The names of the repository/repositories and accession number(s) can be found in the article/Supplementary Material.

## Ethics Statement

The animal study was reviewed and approved by Science Research Department of the Institute of Animal Husbandry and Veterinary Medicine, Beijing Academy of Agriculture and Forestry Sciences.

## Author Contributions

JZ performed the experiments, analyzed the data, and wrote the manuscript. HZ analyzed the data and edited the manuscript. JC and AG collected the samples. HW and QC contributed to project administration. ZY, XZ, and YZ performed the experiments. HL designed the study and reviewed the manuscipt. All authors have read and approved the final manuscript.

## Funding

This research was financially supported by Public Institution Research and Service Project of Beijing Academy of Agriculture and Forestry Sciences (XMS201903) and the Earmarked Fund for Modern Agro-Industry Technology Research System (CARS41-Z01).

## Conflict of Interest

The authors declare that the research was conducted in the absence of any commercial or financial relationships that could be construed as a potential conflict of interest.

## Publisher's Note

All claims expressed in this article are solely those of the authors and do not necessarily represent those of their affiliated organizations, or those of the publisher, the editors and the reviewers. Any product that may be evaluated in this article, or claim that may be made by its manufacturer, is not guaranteed or endorsed by the publisher.

## Supplementary Material

Supplementary Figure S1 Proteins as classified into three main categories by GO analysis: biological process, cellular component, and molecular function in chicken skinless breast meat. The left y-axis indicates the percentage of a specific category of genes in that category. The right y-axis indicates the number of genes in a category.

Supplementary Figure S2 Proteins as classified into six main categories by KEGG analysis: metabolism, genetic information processing, environmental information processing, cellular processes, organismal systems and human diseases in chicken skinless breast meat. The x-axis indicates the number of proteins within that specific category.

Supplementary Table S1 The identified 85,680 match spectra list in skinless breast meat of BYC by TMT.

Supplementary Table S2 The identified 41,768 peptides and 4,935 proteins list in skinless breast meat of BYC by TMT.

Supplementary Table S3 Gene Ontology (GO) analysis of 4,935 proteins in skinless breast meat of BYC.

Supplementary Table S4 Differentially expressed proteins for three comparison groups of D150 vs. D300, D300 vs. D450 and D150 vs. D450.

Supplementary Table S5 GO and KEGG enrichment analysis based on the 664 known DEPs.

Supplementary Table S6 34 and 37 DEPs screened by results of GO and KEGG enrichmen, respectively.

Supplementary Table S7 GO and KEGG enrichment base on 57 DEPs.

## Abbreviations

ABCC4: ATP binding cassette subfamily C member 4
ABCD2: ATP binding cassette subfamily D member 2
ABHD2: abhydrolase domain containing 2
ACAA2: acetyl-CoA acyltransferase 2
ACACA: acetyl-CoA carboxylase alpha
ACADL: acyl-CoA dehydrogenase, long chain
ACADS: acyl-CoA dehydrogenase, C-2 to C-3 short chain
ACAT1: acetyl-CoA acetyltransferase 1
ACOT11: acyl-CoA thioesterase 11
ACOT2 (LOC771753): acyl-CoA thioesterase 2
ACOT4: acyl-coenzyme A thioesterase 1
ACOT7: acyl-CoA thioesterase 7
ACSL1: acyl-CoA synthetase long-chain family member 1
ANXA1: annexin A1
apoA-I: apolipoprotein AI
apoA-IV: apolipoprotein A4
apoB: apolipoprotein B
apoC-III: apolipoprotein C-III
ATP2A2: ATPase sarcoplasmic/endoplasmic reticulum Ca^2+^ transporting 2
ATP2A3: ATPase sarcoplasmic/endoplasmic reticulum Ca^2+^ transporting 3
ATP8B4: ATPase phospholipid transporting 8B4
BP: biological process
BYC: Beijing-You chicken
CALM1: calmodulin 1
CC: cellular component
CPT1A: carnitine palmitoyltransferase 1A
CPT2: carnitine palmitoyltransferase 2
D5D: delta-5 desaturase
D6D: delta-6 desaturase
DECR1: 2,4-dienoyl-CoA reductase 1
DEPs: differentially expressed proteins
DEPs: differentially expressed proteins
ECHDC1: ethylmalonyl-CoA decarboxylase 1
ECI1: enoyl-CoA delta isomerase 1
FA: fatty acids
FABP: fatty acid binding protein
FABP3: fatty acid binding protein 3
FC: fold change
FDR: false discovery rate
GC-MS: Gas chromatography-mass spectrometry
GCSH: glycine cleavage system protein H
GO: Gene Ontology
HADH: hydroxyacyl-CoA dehydrogenase
HADHA: hydroxyacyl-CoA dehydrogenase/3-ketoacyl-CoA thiolase/enoyl-CoA hydratase (trifunctional protein), alpha subunit
HDL: high-density lipoprotein
HSD17B12: hydroxysteroid (17-beta) dehydrogenase 12
IMF: intramuscular fat
KEGG: Kyoto Encyclopedia of Genes and Genomes
LCAT: lecithin:cholesterol acyltransferase
LDL: low density lipoproteins
LPL: lipoprotein lipase
MAPK: mitogen-activated protein kinase
MF: molecular function
MTP: Microsomal triglyceride transfer protein large subunit
MUFA: monounsaturated fatty acid
PECR: peroxisomal trans-2-enoyl-CoA reductase
PECR: peroxisomal trans-2-enoyl-CoA reductase
PHKA2: phosphorylase kinase regulatory subunit alpha 2
PHKB: phosphorylase kinase regulatory subunit beta
PPAR: peroxisome proliferator-activated receptors
PPI: Protein-protein interaction
PPP3R1: protein phosphatase 3 regulatory subunit B, alpha
PRM: parallel reaction monitoring
PUFA: polyunsaturated fatty acid
SCD16: delta-9 desaturase for C16
SCD18: delta-9 desaturase for C18
SFA: saturated fatty acid
SLC25A4: solute carrier family 25 member 4
SLC25A5: solute carrier family 25 member 5
SLC37A4: solute carrier family 37 member 4
SPHK1: sphingosine kinase 1
TECRL: trans-2,3-enoyl-CoA reductase like
TGF-beta: transforming growth factor-beta
TMT-LC-MS/MS: tandem mass tag labeling technology based on liquid chromatography mass spectrometry
TNNC1: troponin C1, slow skeletal and cardiac type
TNNC2: troponin C2, fast skeletal type
VEGF: vascular endothelial growth factor
VTG1: vitellogenin 1
VTG2: vitellogenin 2
YWHAQ: tyrosine 3-monooxygenase/tryptophan 5-monooxygenase activation protein theta.
