# Supplementary figures and images for: Breast Meat Fatty Acid Profiling and Proteomic Analysis of Beijing-You Chicken During the Laying Period
Source: Front Vet Sci. 2022 Jun 15;9:908862. doi: 10.3389/fvets.2022.908862 (PMC9240433; doi:10.3389/fvets.2022.908862)

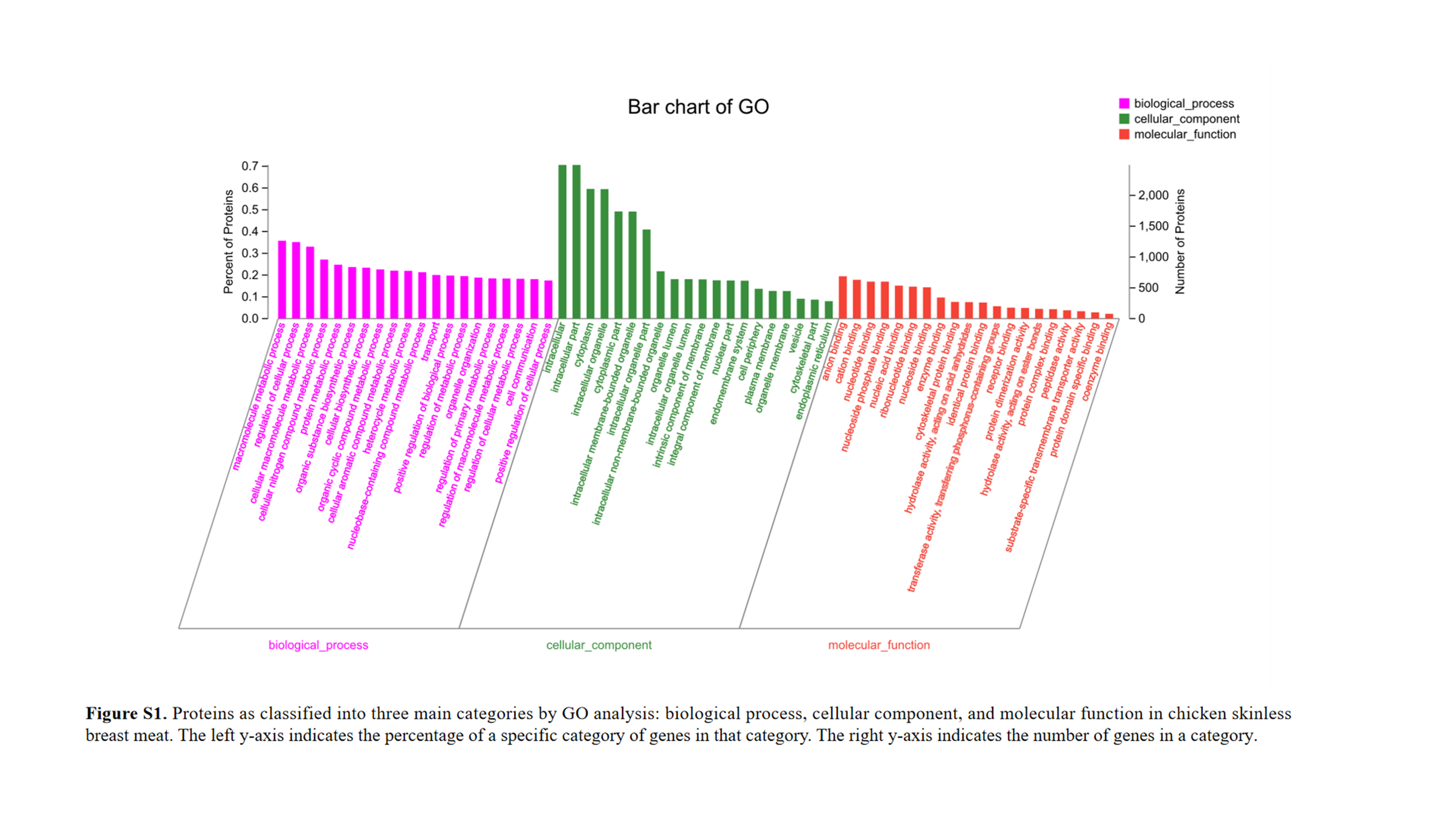

Supplement: Supplementary Figure S1 — Proteins as classified into three main categories by GO analysis: biological process, cellular component, and molecular function in chicken skinless breast meat. The left y-axis indicates the percentage of a specific category of genes in that category. The right y-axis indicates the number of genes in a category. [file Image_1.TIF]

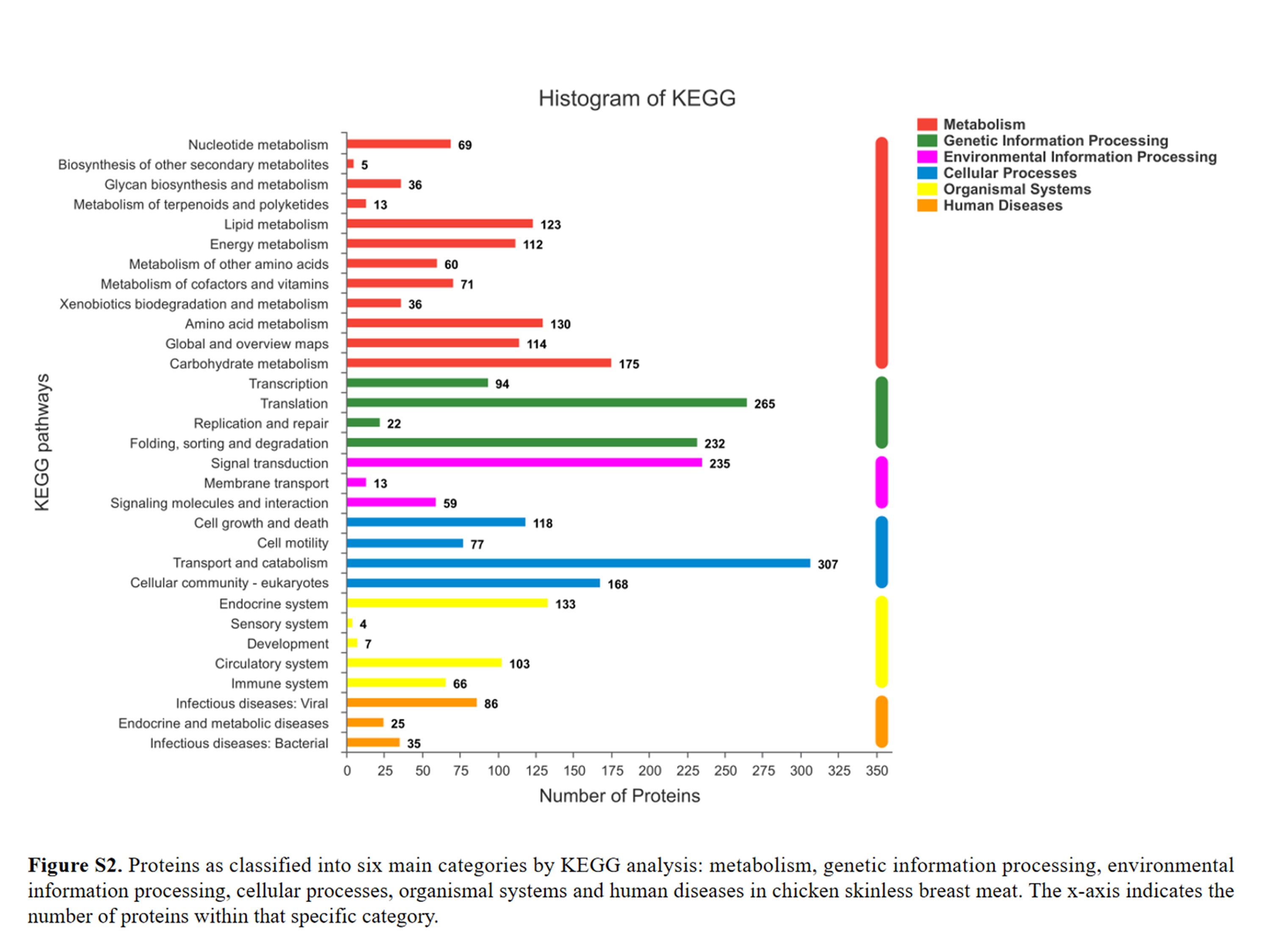

Supplement: Supplementary Figure S2 — Proteins as classified into six main categories by KEGG analysis: metabolism, genetic information processing, environmental information processing, cellular processes, organismal systems and human diseases in chicken skinless breast meat. The x-axis indicates the number of proteins within that specific category. [file Image_2.TIF]
